# Outcomes of Older Adults with Burn Injury: University Clinical Center of Kosovo

**Published:** 2015-07

**Authors:** Shkelzen B. Duci, Hysni M. Arifi, Hasan R. Ahmeti, Violeta K. Zatriqi, Zejn A. Buja, Enver T. Hoxha, Agon Y. Mekaj

**Affiliations:** 1 University Clinical Center of Kosovo, Department of Plastic Surgery, Faculty of Medicine, Prishtina, Rrethi i spitalit p.n. 10000, Kosovo;; 2 University Clinical Center of Kosovo, Department of Pediatric Surgery, Faculty of Medicine, Prishtina, Rrethi i spitalit p.n. 10000, Kosovo;; 3 University Clinical Center of Kosovo, Department of Neurosurgery, Faculty of Medicine, Prishtina, Rrethi i spitalit p.n. 10000, Kosovo

**Keywords:** Adults, Burns, Old, Kosovo

## Abstract

**BACKGROUND:**

Advances in burn care over the past 50 years have brought about remarkable improvement in mortality rates such that survival has become an expected outcome even in patients with extensive injuries. Although these improvements have occurred in all age groups, survival in older adults still lags far behind that in younger cohorts. This study determines the outcomes of older adults with burn injury in University Clinical Center of Kosovo.

**METHODS:**

This is a retrospective study that includes 56 burn patients, older than 60 years who were admitted at the Department of Plastic Surgery, between 1 January 2004 and 31 December 2013. Data processing was done with the statistical package of Stat 3. From the statistical parameters the structural index, arithmetic median, and standard deviation were calculated.

**RESULTS:**

Fifty six burned patient older than 60 years were included during a 10-year period. Of the 56 elderly patients 29 were women and 27 were men with a mean age of 66.7 years (range, 60-85 years). The differences were not statistically significant for both genders regarding the causes of burn injury.

**CONCLUSION:**

Considering the gradual increase of the elderly population in our country based on the data of the Ministry of Public Services, an increase is expected to the incidence of burn injuries in the population of this category of our country.

## INTRODUCTION

Advances in burn care over the past 50 years have brought about remarkable improvement in the mortality rates such that survival has become an expected outcome even in patients with extensive burn injuries. Although these improvements have occurred in all age groups, survival in older adults still lags far behind than in younger cohorts.^1^^-^^5^

Older people are vulnerable to aggressive burns because of skin atrophy, comorbidities, diminished host defense mechanisms and reduced mobility. Older people are more prone to complications of burns and they have longer hospital stay and higher mortality rates.^6^^-^^10^ Worldwide epidemiological research has revealed results and conclusions that reflect unique issues for each country related to burns in the older people.^6^

According to the 2011 registration, the estimated population in Kosovo is 1,800,000.^11^ The high addition of population has caused the high participation of young people in Kosovo. According to the 1981 registration in Kosovo participation of the young people from 0-19 years, it was about 52%, and in elderly population over 65 years of age was 4.6%, and in population of middle age group of 20 to 64 years was 43.4%. Subsequent data, based on estimates for 2000 showed a slow decrease of 42.5% in the young population, and in the elderly population above 65 years; an increase of 5.5% was noticed. The mean age of the population of Kosovo in 2000 was 22.2 years, indicating that the Kosovo population is young one.^12^

However, there is dearth of data about the consequences of burns in older people in Kosovo. This retrospective study investigated burn patients older than 60 years who were admitted to our hospital, Department of Plastic Surgery during a period of 10 years to assess the epidemiology and outcomes of burn injuries. 

## MATERIALS AND METHODS

This is a retrospective study that included 56 burn patients of older than 60 years who were admitted at the Department of Plastic Surgery, between January 1, 2004 and December 31, 2013. Data about the gender, age, seasonal variation, location, cause, burn size (TBSA), treatment of burn-related injuries, duration of hospitalization and mortality were recorded. The data was collected and analyzed from the medical records.

The policy at our intensive care unit is to admit any burned patient older than 60 years who met any of the American Burn Association major burn injury criteria: (i) Burn in 10% of total body surface area (TBSA), (ii) Localized deep burn affecting ≥ 5% TBSA, (iii) Facial burn, (iv) Suspected inhalation injury, (v) Hands, feet or perineum burns, (vi) Chemical or electrical burns, and (vii) Associated fracture or chronic illness.

All patients received first aid in the reception room of the emergency unit. There, intravenous access was established, the respiratory airway was cleared and endotracheal intubation was performed if necessary. The evaluation of patients was done by a plastic surgeon regarding the treatment of patients in Intensive Care Unit or Department of Plastic Surgery since a burn center has not been established in our country yet. Each elderly patient was evaluated on an individual basis, and excision or grafting was not performed in situations where a previous chronic illness was identified, such as uncontrolled diabetes mellitus or aggravated cardiovascular diseases. 

Data processing was done with the statistical package of Stat 3. From the statistical parameters; the structural index, arithmetic median, and standard deviation were calculated. Data testing was done using the test and the differences were considered significant if *p<0.05.*

## RESULTS

In this study, 56 burned patients older than 60 years were included in a 10-year period. Of the 56 elderly patients, 29 were women and 27 were men with a mean age of 66.7 years (range, 60-85 years) (Table 1). The corresponding mean ages of females and males were 67.3 and 66.1 years, respectively.

**Table 1:** Case distribution by age and gender

**Age groups (years)**	**Gender**		**Total**	
	**Male**	**Female**	**n**	**%**
60-65	16	11	27	48.2
66-70	5	11	16	28.5
71-75	3	4	7	12.5
76-80	2	2	4	7.1
81-85	1	1	2	3.5
Total	27	29	56	100.0
Mean±standard deviation Range			66.7±10.0 years 60-85 years	

**Fig. 1:**
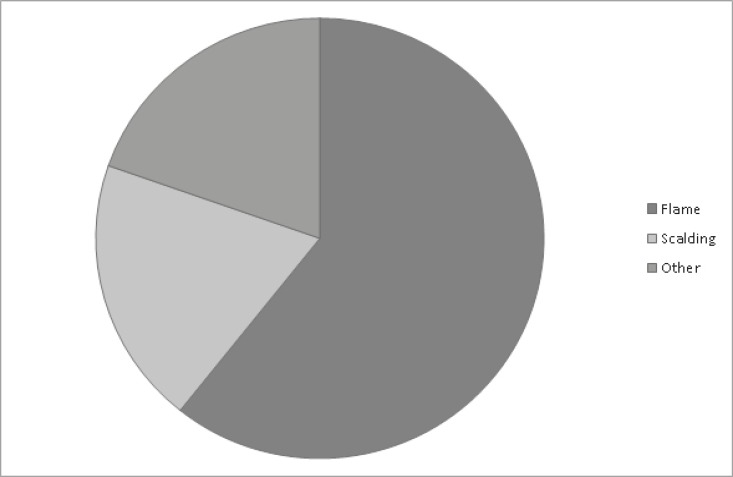
Patients suffered from scalding

Regarding the season, the incidence of burns was higher in winter with 21 patients (37.5%), followed by spring with 19 patients (33.9%), autumn with 9 patients (16%), and summer with 7 patients (12.5%). Thirty-two patients (57.1%) lived in rural areas while 24 patients (42.8) lived in urban regions. The home was the most common location where burns occurred. Fire was the most common factor involved in the majority of these cases with 34 patients (60.7%), 11 patients (19.6%) suffered from scalding (Figure 1). 

**Fig. 2:**
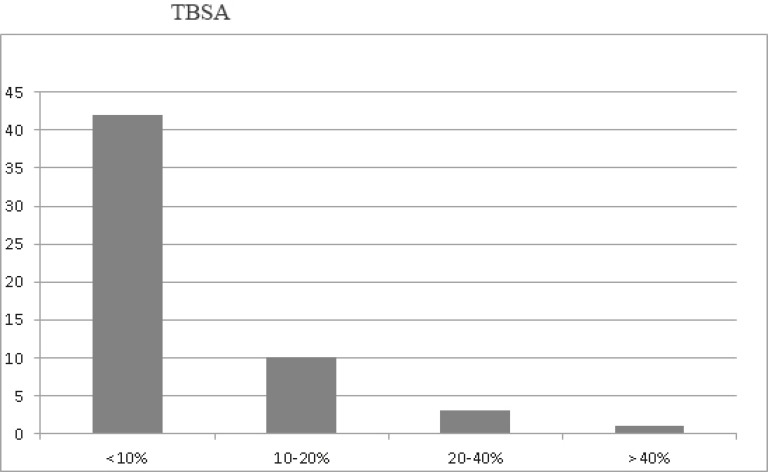
Patients sustained burns based on TBSA

Hot water was the most common factor involved in the majority of these cases. Five patients (8.9%) suffered electrical burn injuries. Three patients suffered burns caused by hot object and all these patients were with diabetes mellitus. Also two patients suffered from chemical burn and one patient suffered from radiation burn (Table 2). Forty two patients (75%) sustained burns involving less than 10% TBSA; 10 (17.8%) sustained burns of 10-20% TBSA; 3 (5.3%) sustained burns of 20-40% TBSA; and 1 sustained burns of greater than 40% TBSA (Figure 2).

**Table 2:** Causes of burns by Sex

**Causes of burns**	**Male N (%)**	**Female N (%)**	**Total N (%)**	**P value**
Flame	20 (35.7%)	14 (20%)	34 (100.0%)	
Scalding	3 (5.3%)	8 (14.2%)	11 (100.0%)	
Electrical burns	4 (7.1%)	1 (1.7%)	5 (100.0%)	
Hot objects	2 (3.5%)	1 (1.7%)	3 (100.0%)	
Chemical	2 (3.5%)	----	2 (100.0%)	P=0.6029
Radiation	-----	1 (15.4%)	1 (100.0%)	

Thirty eight (67.8%) of the 56 admitted patients were treated with early tangential excision followed by skin grafting, 3 patients (5.3%) underwent fasciotomy and escharotomy due to compartment syndrome, 1 patient with diabetes underwent amputation of the digits of lower extremity remaining the patients were treated with medical therapy and supportive measures. The average hospital stay for the 56 admitted cases was 13.8 days, and the range was 1-59 days. One case was transferred for treatment in Turkey. One patient from 56 elderly patients died and was with TBSA above 40%.

## DISCUSSION

Burn injury constitutes a significant epidemiological problem in older adults.^1^ The elderly burn patients demonstrate greater mortality and the aged survivors show slower recoveries, increased length of hospital stay and suffer more complications than their younger counterparts.^14^ In this retrospective study, 56 burned patient older than 60 years were included in the 10-year period. Of the 56 elderly patients, 29 were women and 27 were men. We have a slight predominance of women compared with other studies conducted by Simesk et al. regarding burns on the elderly patients.^6^ Such a dominance of females can be explained by the fact because in our country our grandparents live together with their children while elderly women-prepare meals in kitchen for family especially in rural areas while son and bride are working at long distance cities. For this reason, these women are more vulnerable to burns caused in the household with other preconditions (dementia, weakened vision etc.).

The most frequent cause was flame burns followed by scalding and electrical burns and occurred in winter and spring. This is probably related by the fact that our country after 1999 emerged from the war and our population was confronted with frequent reductions of electricity, especially during the winter season due to overloading of the electricity network; at the same time, our population within households were forced to find other alternative for electricity source (aggregates with fuel for the production of electricity, gas canisters for the preparation of food etc.). As a result, we have this dominance of burns in elderly patients during winter season.^11^

Three patients suffered burns caused by hot objects. All these burns due to hot objects have occurred during the winter season and all this patients were with diabetes. The reason for such burns is heat of the lower extremities with hot objects in diabetics to which as we know, we have loss of sensitivity to detect the low and high temperature due to the development of diabetic angioneuropathies.

Thirty-two patients (57.1%) lived in rural areas while 24 patients (42.8) lived in urban regions. There may be several reasons for this predominance. First, rural population in our country lives in significantly poorer conditions than the urban population. They usually use coal burning stoves and electric stoves for heating of water, milk and soup. Secondly, most urban families have 2 to 3 children and they are most likely to be more vigilant compared with rural families who usually have more than one child or both parents may have to work in a distant city. This leaves the children to be cared by their grandparents who have additional load with other works around the house which makes more vulnerable for injuries of this nature.^15^

Thirty eight (67.8%) of the 56 admitted patients had full thickness burn injuries and were treated with early tangential excision followed by skin grafting, 3 patients (5.3%) underwent fasciotomy and escharotomy due to compartment syndrome, the remaining in the group was treated with medical therapy and supportive measures. Of the 56 admitted patients, 5 cases suffered from diabetes from which one case was complicated with the cellulitis of the lower extremities and another case with gangrene of the toes of the lower extremities and underwent surgical amputations of all toes in the metatarsal joint. Most of these patients were hospitalized with delay in our institution usually after 72 hour due to incorrect assessment of burned surfaces in primary medical services.

The average hospital stay for the 56 admitted cases was 13.8 days, and the range was 1-59 days. A similar study by Palmieri et al. reported an average hospital stay of elderly patients of 11.2 days, which is approximately similar to our study.^16^ One case was transferred for treatment to Turkey, and we do not have data concerning the outcome of this patient. In this retrospective study, we found a rather lower frequency of mortality in elderly burn patients requiring hospitalization. One patient from 56 elderly patients died and was with TBSA above 40%. Such a lower incidence of burns in this category of population and mortality probably is related due to several reasons. 

Hence, regarding subsequent data for the year 2000; the mean age of the population of Kosovo was 22.2 years, indicating that Kosovo is dominated by a young population.^12^ Secondly, such a lower mortality probably is due to lower incidence of major burns and appropriate resuscitation of these patients in the acute phase of burn injuries in our institution. 

Based on the results of this study, we can conclude that (i) Considering the gradual increase of the elderly population in our country based on the data of the Ministry of Public Services, an increase is expected in the incidence of burn injuries in the population of this category in our country, (ii) Most of these patients were hospitalized with delay in our institution usually after 72 hours due to incorrect assessment of burned surfaces in primary medical services. Therefore, complication and morbidity increased especially in patients with diabetes and other co-morbidities. As a consequence, we need to organize special training programs for practitioners in primary care services through which they will be familiarized with this kind of trauma, treatment and complications due to delayed referral, and (iii) Ongoing effective health prevention programs also necessitate focusing on prevention with the aim of a reduction of burn events in this category of population.

## CONFLICT OF INTEREST

All authors declare no financial and personal relationships with other people or organizations that could inappropriately influence (bias) their work. No potential conflicts of interests were disclosed. This study does not have any sponsor and is own work of authors.

## Note

Please cite this paper as:

Duci SB, Arifi HM, Ahmeti HR, Zatriqi VK, Buja ZA, Hoxha ET, Mekaj AY. Outcomes of Older Adults with Burn Injury: University Clinical Center of Kosovo. World J Plast Surg 2015;4(2):153-158.
